# Modeling Red Blood Cell Viscosity Contrast Using Inner Soft Particle Suspension

**DOI:** 10.3390/mi12080974

**Published:** 2021-08-18

**Authors:** Alžbeta Bohiniková, Iveta Jančigová, Ivan Cimrák

**Affiliations:** 1 Research Centre, University of Žilina, 010 26 Žilina, Slovakia; alzbeta.bohinikova@rc.uniza.sk (A.B.); ivan.cimrak@fri.uniza.sk (I.C.); 2 Cell-in-Fluid Biomedical Modelling and Computations Group, Faculty of Management Science and Informatics, University of Žilina, 010 26 Žilina, Slovakia

**Keywords:** viscosity, dissipative particles, blood cells, computational modeling, rheology

## Abstract

The inner viscosity of a biological red blood cell is about five times larger than the viscosity of the blood plasma. In this work, we use dissipative particles to enable the proper viscosity contrast in a mesh-based red blood cell model. Each soft particle represents a coarse-grained virtual cluster of hemoglobin proteins contained in the cytosol of the red blood cell. The particle interactions are governed by conservative and dissipative forces. The conservative forces have purely repulsive character, whereas the dissipative forces depend on the relative velocity between the particles. We design two computational experiments that mimic the classical viscometers. With these experiments we study the effects of particle suspension parameters on the inner cell viscosity and provide parameter sets that result in the correct viscosity contrast. The results are validated with both static and dynamic biological experiment, showing an improvement in the accuracy of the original model without major increase in computational complexity.

## 1. Introduction

Blood is a multi-component suspension and consists mostly of plasma (~55%), red blood cells (RBCs, ~45%) and white blood cells and platelets (~1%). Due to their high content, RBCs play major role in flow dynamics and rheological properties of blood. A healthy RBC has a biconcave shape with a diameter 6–8 μm and thickness 2 μm [1]. The RBC membrane consists of a lipid bilayer and a spectrin network attached to the inner side of the bilayer. The cytoplasm contains mostly hemoglobin, a protein responsible for oxygen transfer. This protein causes higher viscosity of the inner cytoplasm compared to the outer plasma, which is referred to as viscosity contrast.

The blood flow in large vessels is mostly driven by a uniform shear flow that occurs near the vessel walls or by the fairly uniform flow in the central parts of the vessels. On the contrary, in vessel bifurcations and in small arterioles and microcapilaries with diameters comparable to the size of the cells, the parabolic velocity profile introduces non-uniformity and the flow patterns differ significantly from those in large vessels.

The shape of the red blood cell in large vessels is mostly close to the relaxed biconcave shape or to prolonged ellipsoidal shapes, with dynamic patterns such as tumbling and tank-treading [2,3,4,5]. In capillaries, the shape patterns are richer. Different flow conditions result in cup-like parachute shapes at the vessel center, elongated slipper shapes at an off-center position, bullet-like shapes in very narrow vessels, snaking dynamics—a periodic cell swinging around the tube center, or tumbling trilobe state at large flow rates and low confinements [6].

Besides the flow conditions and the elasticity of the membrane, an important parameter influencing the cell dynamics is the viscosity contrast Λ. It has been shown that Λ significantly affects the RBC behavior in simple shear flow [7,8,9,10]. Only recently, several studies have confirmed that the viscosity contrast is important in microcapillary flow [6,11,12,13,14]. In [6], the authors study the dependence of the RBC shape and dynamics on the viscosity contrast in tube flow. They provide state diagrams of RBC dynamical states for various viscosity contrasts and wide ranges of flow rates and tube diameters. They conclude that the region of parachute shapes is significantly larger for Λ=1 in comparison to Λ=5. In another study [13], many-cell simulations show an increasing difference between relative apparent viscosity for Λ=1 and Λ=5 with increasing Reynolds number. This indicates that the viscosity contrast plays a more important role as the flow shear rates increase.

An interesting result concerns the cell free layer (CFL). While in works [11,13] the authors conclude that the viscosity contrast influences the thickness of the CFL only very weakly, if at all, the opposite conclusion has been drawn in [14] where the authors claim that at 10% hematocrit, the viscosity contrast is not negligible when calculating the CFL thickness. They study small arteriole flows (20–40 μm) with a hematocrit of 10–20% and a physiological viscosity contrast, which is Λ=5 on average [15,16].

The viscosity contrast has proven to play an important role in microcirculation. In this work, we employ a novel approach to taking the viscosity contrast into account. We extend the existing red blood cell model based on lattice-Botzmann method (LBM) for governing the fluid and spring network for governing the elasticity of cell membrane [17,18]. We include dissipative particles (DPs) inside the cell as a coarse-grained hemoglobin model to increase the viscosity of the resulting suspension inside the cell. We analyse the influence of suspension properties on its effective viscosity. We model two scenarios to measure the suspension viscosity. The results from these two computational experiments are then used in the RBC model with DPs. The new model is validated using biological data from the static RBC stretching and the dynamic release [19].

## 2. Viscosity Contrast in Other Red Blood Cell Models

Many of the recent simulation studies for simplicity assume the viscosity contrast Λ=1 [20,21,22,23]. For lower shear stresses and for the flows with no small confinements, it is a reasonable assumption that can be deduced from conclusions of [11,13].

There are, however, models that assume non-unit viscosity contrast. The continuous model in [14] for example treats the coupled problem governed by Navier–Stokes equations that conserve momentum both in the fluid and in the membrane sub-domains. Computational domain is divided into fluid and cell with the stress balance to be satisfied at the boundary of fluid and cell. The cell is modeled as a 2D hyperelastic membrane with Skalak model. To solve the coupled fluid–solid problem, they utilize an Immersed Finite Element Method. The viscosity contrast is naturally incorporated by different fluid properties inside the cell domain. This method requires re-meshing of the computational domain when the cell deforms.

The method in [6,11] is based on smoothed dissipative particle dynamics. Here, the fluid consists of fluid particles which interact through conservative, dissipative and random forces. The cytosol inside the RBC is separated from the outside fluid (plasma) by the layer of membrane particles. The number of membrane particles is typically around 3000 and the density of the fluid particles can be set from 9 to 12 per unit volume (1 μm${}^{3}$) [6,11]. The viscosity of the inner and outer fluid is achieved by frictional dissipative force between neighbouring particles. Therefore, the viscosity contrast can be increased simply by taking two different magnitudes of the dissipative forces for the inner and outer particles. The elasticity of the membrane is achieved by using a spring network of particles with elastic moduli: stretching, bending, local and global area and volume modulus. The elastic forces are computed by minimization of total potential energy of the particle system.

The model in [13] consists of coupled lattice-Boltzmann and Immersed Boundary method (IBM). The blood plasma is represented as a continuous incompressible Newtonian fluid that is computed using LBM with a fixed lattice with 19 directions providing second order accuracy for fluid computations. The cells are represented as spring-network meshes with elastic properties defined by the total potential energy similar to that of the model presented in [6,11]. The fluid and the membrane are coupled using a validated immersed boundary method [22]. The fluid viscosity in LBM is controlled by a relaxation parameter τ and in general, this parameter can be variable in space ensuring different viscosities at different locations. It is important to carefully handle the discontinuity when considering two distinct viscosities for inner and outer membrane fluid. To introduce a viscosity contrast into such model, one needs to keep track of the fluid nodes residing inside the RBC membranes. These membranes are represented as closed surface triangular meshes which enclose lattice nodes of the fluid grid. For these grid nodes, the relaxation parameter τ of the LBM method can be altered to express the increase in local viscosity. In [22], determining whether a lattice point is inside an RBC was done using the ray-casting algorithm, where for a point inside or outside a polygon a ray is cast and the number of membrane intersections is counted. Odd number means the point is an interior point. Although the complexity of such algorithm is high, the authors have optimised the performance to ensure the increase in typical runtime is less than 25%.

## 3. RBC Model and the Concept of Dissipative Particles

We work with an RBC model that we previously developed and introduced in [17,18]. The model consists of two principal components: the fluid and elastic objects immersed in it. For the fluid, we use the lattice-Boltzmann method [24]. The three-dimensional space is discretized with a fixed lattice. Fictive particles can move and collide over the edges and the diagonals of this lattice, representing coarse-grained fluid. From the density and movements of these particles, one can express the velocity, density and other properties of the fluid in each time step on a fixed lattice grid.

**Figure 1:**
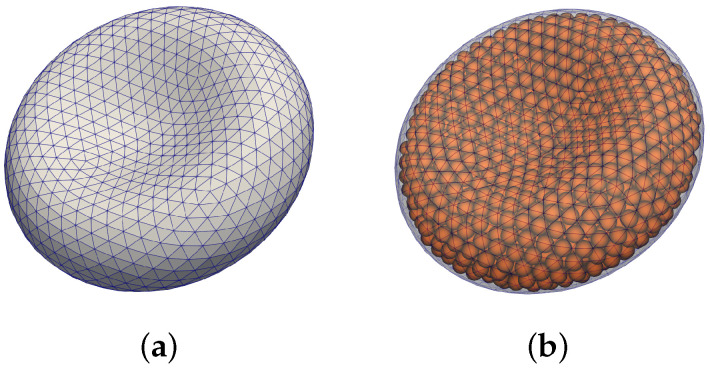
Visualization of RBC model. (**a**) triangular mesh modelling the membrane, (**b**) dissipative particles included inside the membrane.

The second component is a triangular mesh representing a closed surface of the cell membrane, see Figure 1a. The elastic properties of the object are represented with different types of force-like bonds between neighboring mesh points. This way, the deformation of the object changes the relaxed distances between the mesh points and this induces forces acting against the change at the corresponding mesh points.

Unlike in the IBM described above, the fluid and the elastic objects are coupled via a dissipative force here. Forces induced by the cell deformation enter the lattice-Boltzmann equation. Analogously, fluid forces are also transferred to the object’s mesh points. This method has been known as Immersed Boundary via Dissipative Coupling (IB-DC).

The two distinct meshes—the lattice mesh for the fluid and the triangular mesh for the cell—overlap, which results in uniform viscosity for the fluid inside and outside the cell. One solution for introducing the viscosity contrast in LBM was described above in combination with the IBM method [13]. Another solution, presented here, is to introduce the viscosity contrast by mimicking the biological essence of cytosol content, the hemoglobin proteins.

The major component of RBC is water (721 mg mL${}^{-1}$ RBC), which is twice the mass of total RBC protein (371 mg mL${}^{-1}$ RBC). Hemoglobin makes up 95% of total RBC protein [25]. This may suggest that including hemoglobin into the model of RBC would lead to correct modelling of the viscosity contrast.

Hemoglobin is a protein that folds into globular structures [26]. There are no substantial holes and almost no water molecules in the protein interior. As a consequence, the proteins are rigid structures. Their spheroidal shape was confirmed by micrograph [27] to be of 6 × 6 × 5 nm${}^{3}$ as presented in Table 4 in [28].

In this work, we employ a coarse-graining method called dissipative particle dynamics (DPD). Since modelling each individual hemoglobin molecule is computationally too expensive (rough calculation would lead to $10^{8}$ hemoglobin molecules in one cell), we represent a virtual cluster of hemoglobin molecules as one soft dissipative particle (DP). The dissipative forces are included in the particle interactions due to the eliminated degree of freedom during the coarse-graining procedure [29]. Similar approach for modelling of the aligned hemoglobin polymer in sickle-cell RBCs was presented in [30,31]. The vizualization of the model with particles is depicted in Figure 1b.

## 4. Practical Considerations for Model Implementation

The typical number of mesh points in the triangular mesh of IB-DC model can vary. At least 200 mesh points are needed to capture the cell shape properly but with increased demand on the accuracy, the mesh can contain up to few thousand mesh-points. The aim is to keep the complexity of the model comparable and thus the number of inner particles should not exceed couple thousand.

The coarse-graining procedure clusters a large number of hemoglobin molecules into one dissipative particle. Although the hemoglobin molecules are rigid, the virtual clustering causes the particle to be soft. It is thus modelled as a non-dimensional particle with radius $r_{h}$ interacting with other particles by two kinds of forces: conservative and dissipative. The classical DPD approach also includes random forces but on the scale we are interested in, the Brownian motion is not of the interest and thus we omit the random component. The conservative forces depend on the position of the particles and are purely repulsive, while the dissipative forces depend on the relative velocity between the particles.

*Conservative forces* We consider two different repulsive potentials with cutoff distance being twice the radius of the DPs. 

The first is the soft-sphere potential leading to the repulsive forces $F_{soft}$ between two particles at distance *d*

$$F_{soft}=\left\{\begin{array}{cc} a\cdot n\frac{1}{d^{n+1}} & \;d\leq c_{soft}\\ 0 & \;d>c_{soft} \end{array}\right.$$

where the parameters a,n define the magnitude and steepness of the potential and $c_{soft}=2r_{h}$ its cutoff distance. Note that the soft-sphere forces blow up at zero particle distance and are not continuous at the cutoff distance.

The second potential is a hat potential defined as

$$F_{hat}=\left\{\begin{array}{cc} F_{max}\left(1-\frac{d}{c_{hat}}\right) & \;d\leq c_{hat}\\ 0 & \;d>c_{hat} \end{array}\right.$$

where $F_{max}$ defines the maximal repulsive force when the two particles overlap which linearly decreases to zero at cutoff distance $c_{hat}=2r_{h}$.

*Dissipative forces* For modelling of the viscosity, the dissipative forces consist of two components, one that aligns with the particle-particle line and one that is perpendicular to it:

$$F_{DPD}^{\|}=\left\{\begin{array}{cc} \gamma_{\|}\langle {\vec{r}}_{ij},{\vec{v}}_{ij}\rangle {\vec{r}}_{ij} & \;d\leq c_{DPD}\\ 0 & \;d>c_{DPD} \end{array}\right.$$

$$F_{DPD}^{⊥}=\left\{\begin{array}{cc} \gamma_{⊥}\left({\vec{v}}_{ij}-\langle {\vec{r}}_{ij},{\vec{v}}_{ij}\rangle {\vec{r}}_{ij}\right) & \;d\leq c_{DPD}\\ 0 & \;d>c_{DPD} \end{array}\right.$$

where 〈·,·〉 denotes scalar product, ${\vec{r}}_{ij}$ is the unit vector between two particles i,j, ${\vec{v}}_{ij}$ is the relative velocity and $c_{DPD}=2r_{h}$ is the cutoff radius. Although the dissipative forces have two distinct contributions, parallel with and normal to ${\vec{r}}_{ij}$ and can in principle be scaled differently, we use a common multiplicative parameter $\gamma_{DPD}$ for both of them, so $\gamma_{DPD}=\gamma_{\|}=\gamma_{⊥}.$

The number of particles inside an RBC can vary and depends on the $r_{h}$ and the desired solid-volume fraction ϕ. Both $r_{h}$ and ϕ influence the viscosity of the resulting suspension.

To ensure that the DPs stay within the cell membrane, we use another soft-sphere potential defining the repulsive forces $F_{soft}^{*}$ between the membrane mesh and the DPs. The definition of $F_{soft}^{*}$ is analogous to $F_{soft}$ with parameters $a^{*}$ and $n^{*}$. The cutoff radius for this potential is denoted by $c_{soft}^{*}$ and its fine-tuning depends on the size of edges in the triangular membrane mesh. The cutoff radius $c_{soft}^{*}$ must be large enough so that the DPs cannot slip through any of the triangles. A reasonable value of $c_{soft}^{*}$ is the average length of the edges in the relaxed mesh. A similar force is also used for the repulsive interaction between the membrane and the outer suspension in Section 5.2.2 with parameters given in Table A4.

Once we decide the size and number of particles, we randomly seed them inside a rhomboid inscribed into the cell. We turn on the repulsive forces $F_{soft}$ between the DPs and $F_{soft}^{*}$ between the DPs and the triangular mesh and we let the particles relax into equilibrium. An increase in $r_{h}$ or ϕ is needed if they do not fill up the whole inner region of the cell. The resulting equilibrium positions are saved and used later for subsequent simulations as initial seedings.

## 5. Viscosity of Particle Suspensions

Particles suspended in liquid increase its effective viscosity. There are different phenomena for suspensions of rigid particles and soft particles.

### 5.1. Suspensions of Spheres or Colloids

For rigid spheres, it is possible to derive analytical formulas that relate the viscosity of suspension to the underlying solid volume fraction. If the particle concentration ϕ is very low (≲0.1 volume fraction), the resulting viscosity can be expressed as

(1) $$\eta =\eta_{0}\left(1+\frac{5}{2}\phi +O\left(\phi^{2}\right)\right),$$

where $\mu_{0}$ is the viscosity of the underlying fluid [32]. The corrected version for a dilute emulsion of viscous droplets was derived in [33]

(2) $$\eta =\eta_{0}\left[1+\frac{5}{2}\phi \left(\frac{\mu^{\prime }+\frac{2}{5}\mu }{\mu^{\prime }+\mu }\right)+O\left(\phi^{2}\right)\right],$$

where $\mu^{\prime }$ is the viscosity of the droplet fluid.

The Formula (1) was later adapted [34,35] in the case of pure straining without effect of Brownian motion for spheres of the same size with total volume fraction from 0.15 to 0.2 as

(3) $$\eta =\eta_{0}\left(1+\frac{5}{2}\phi +7.6\phi^{2}\right).$$

For even higher volume fractions there is an analytical expression [36] that takes into account also two other parameters

(4) $$\eta =\eta_{0}{\left(1-\frac{\phi }{\phi_{max}}\right)}^{-\left[\eta \right]\phi_{max}},$$

where $\phi_{max}$ is the maximum possible solid fraction and (η) is a parameter related to particle shape (with value of 2.5 for spheres). The maximum solid fraction is $\phi_{max}=0.74$ for a regular packing and approximately $\phi_{max}=0.64$ for a random packing of spheres [37].

While the formulas for rigid spheres are not easily applied to soft-sphere suspensions that exhibit much richer rheological properties, they can serve as an upper bound.

For colloidal suspensions [38,39], the models treat particles as hard or soft spheres with lubrication forces suspended in a fluid. The modelling of particle behaviour involves tracking their position, mass and rotational inertia that enter the Newton motion equations together with lubrication forces. The results presented in the above works show nonlinear dependence of the suspension viscosity on the volume fraction and the shear rate.

In Ref. [40], the authors use simulations to establish a relation between the structure of soft colloids and their macroscopic properties in the flow. They study colloids consisting of linear, star-shaped or dendrimer polymers.

When modeling the colloids, the particles in the model typically represent individual physical objects. In our case, the molecules of hemoglobin are too small to be treated individually and we coarse-grain thousands of them into one particle.

### 5.2. Computational Viscosity Measurements

Looking at the biological viscosity values, listed in Table 1 and taking into account the large variance of whole blood viscosities (depending on the shear rate under which it was measured), it is generally concluded that the RBC cytosol is about five times as viscous as the blood plasma. Our aim is to determine parameters of DPs so that the resulting medium will reach those values. To this end, we provide a study analysis of the influence of those parameters on the suspension viscosity.

To measure the viscosity of a particle suspension, we design two computational experiments that resemble the laboratory experiments for measuring the viscosity. The classical viscometers employ either rotational shear or falling of a sphere principle. In both approaches, the fluid exerts drag force either on a rotating plate or on a sinking ball. Simulation parameters can be found in the Appendix A, Table A1, Table A2, Table A3 and Table A4.

#### 5.2.1. Shear Flow between Two Plates

To mimic the shearing viscometer, we model a simple shear flow between two plates. In Couette (shear) flow, the velocity varies linearly from zero at the bottom to $u_{t}$ at the top. This means that the shear rate defined as u/h, where *h* is the height, is constant.

The relationship between the applied force per unit area and the (dynamic) viscosity is as follows:

$$\frac{F_{shear}}{A}=\eta \frac{u}{h}.$$

While this relationship between shear stress and viscosity is linear and can be used as a first approximation, it is known that blood is a shear-thinning fluid, meaning that its viscosity decreases as the shear stress increases, due to blood cells aligning and sliding over each other.

In this computational experiment, we apply the given force $F_{shear}$ per area *A*, measure the shear rate u/h and calculate the resulting viscosity. We can do this for various soft sphere suspensions and determine the parameters that give the viscosity we need.

**Figure 2:**
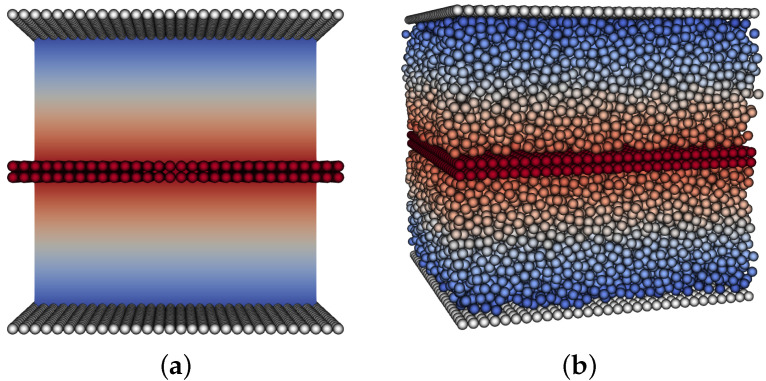
Modified computational shear flow experiment. Both upper and lower boundaries have zero no-slip conditions, with force applied to a layer of particles in the middle. This results in two mirrored sections with linear velocity profiles. (**a**) no particles, (**b**) particle volume fraction ϕ=0.5.

In order to apply the force per unit area on the moving upper plate, we modify the experiment, Figure 2, and mirror the geometry. Here, both upper and lower boundaries are stationary and there is a layer of particles in the middle that has a force applied in the *x*-direction. In order to validate this viscosity measurement, we tested an empty channel and recovered the same viscosity as was set as the fluid parameter.

To measure the viscosity of the simulated particle suspension, we also fix a layer of stationary particles at the bottom and top walls to avoid slip issues at the boundaries.

The shear rates that we want to examine range from 0 to 200 s${}^{-1}$, since these levels of rates were measured for red blood cells in biological experiments [47,48].

#### 5.2.2. Drag Force Experiment

**Figure 3:**
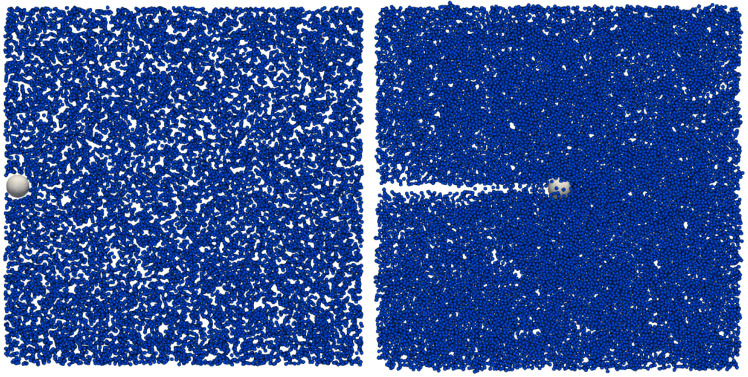
Initial position of a sphere in a drag force computational experiment (**left**). A horizontal force $F_{drag}$ is applied to the sphere, eventually resulting in a steady terminal velocity (**right**). To visualise the cell movement in the suspension, only a slice (width ±1 μm from the central x−y plane of the channel) of the suspensions is rendered. Size of the channel 96 × 96 × 96 μm${}^{3}$, cell radius R = 3 μm.

To make a computational analogue to the falling-sphere experiment, we apply force $F_{drag}$ in the direction of *x*-axis to have a sphere moving in a stationary fluid (suspension of soft DPs), see Figure 3.

The viscosity is then measured, using the Stokes Law, as

(5) $$\eta =\frac{F_{drag}}{6\pi Rv}$$

where η is the dynamic viscosity, $F_{drag}$ is the drag force, *R* is the radius of the sphere and *v* is the (terminal) flow velocity.

This relation holds in an ideal infinite domain. For practical computational purposes, we cannot use periodic boundary conditions since this leads to ever-increasing velocity of the sphere. On the other hand, by imposing solid boundaries, i.e., zero velocity on the boundary, we introduce an artificial boundary effect and the terminal velocity is lower than theoretical. In order to compensate for this, we first evaluate the motion of the sphere in free fluid without particles. In this case, the fluid viscosity is known and thus we can compute the terminal velocity from (5). Given $F_{drag}=0.4$ nN, *R* = 3 μm, number of mesh nodes = 304, and η = 1.2 mPas, we calculate the terminal velocity $v_{\infty }$ = 5.894 mms${}^{-1}$ in infinite domain. We compared different sizes of cube simulation boxes *L* to measure the boundary effects, Table 2.

The boundary effect is calculated as relative deviation of computed terminal velocity from the theoretically predicted value

(6) $$\frac{v_{term}-v_{\infty }}{v_{\infty }}.$$

**Figure 4:**
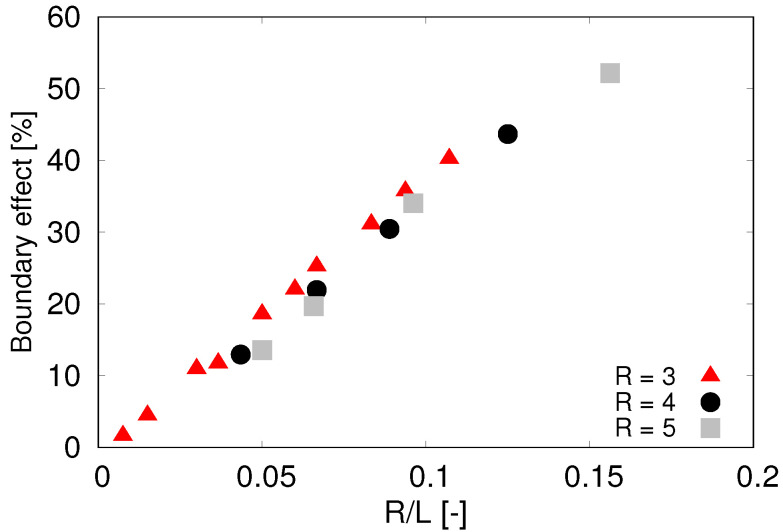
Boundary effect—relative deviation of computed terminal velocity from the theoretically predicted value. Sphere radius *R* given in μm.

Testing various sizes of the sphere revealed that the effect of the boundary is not fixed to the size *L* of the channel but rather to the ratio R/L of the cell radius and the size of the cross section of the channel. The complete analysis of the boundary effect is presented in Figure 4 which gives us guidelines how to correct the simulated terminal velocities depending on the ratio of object size and size of the simulation box.

We also tested a range of drag forces 0.05–0.4 nN and two values of fluid viscosity 0.7, 1.2 mPas. These variations had only insignificant impact on the terminal velocity.

Due to the shear thinning properties of particulate suspensions, we need to compare results corresponding to the same shear rates. While in the experiment with two parallel moving plates, the shear rate is explicitly given by the velocity of the moving plates, in the drag force experiment, the shear rate is computed from the fluid velocity gradient near the top and bottom pole of the sphere, assuming horizontal movement of the sphere in the positive *x*-direction.

The computational complexity of large dense simulations restricts the number of dissipative particles for this computational experiment and implies a lower bound on the radius of the particles. In order to maximize the domain size which reduces the correcting factor depicted in Figure 4, we performed most computational experiments with particle size $r_{h}$ = 0.5 μm. For the computational domain 100 × 100 × 100 μm${}^{3}$ with particle-volume fraction ϕ=0.5 this accounts for ∼10${}^{6}$ particles. A decrease to $r_{h}$ = 0.25 μm would lead to eight-fold increase in number of particles with the same solid-volume fraction.

### 5.3. Determining Appropriate Particle-Volume Fraction

Our aim is to reach the viscosity ratio of at least 4. In relation (4), one can take the values $\phi_{max}=0.67,\left(\eta \right)=2.76$ from [36] and conclude that we need at least ϕ=0.35 to have the viscosity contrast close to 4.

Computational experiments for purely conservative forces ($\gamma^{⊥}=\gamma^{\|}=0$ that correspond to no phenomenological viscosity effects in particle suspension) show that it is not possible to have the viscosity ratio higher than two. It is thus clear that we need to also include the dissipative forces. For such forces to have any effect, the pairs of particles need to be closer than the cutoff distance. This fact thus creates a lower bound for ϕ. Indeed, if the particle-volume fraction is too low, the particles do not touch and their distance is larger than cutoff distance for dissipative forces and therefore no dissipation is present.

On the other hand, too high value of ϕ would make the inter-particle distances smaller than cutoff (effectively meaning an overlap of the DPs) which in turn, in combination with conservative forces, would create a constant tension and make the inner suspension too stiff for a cell to relax back to its original shape.

To determine the appropriate value of particle-volume fraction ϕ, we performed the following set of computational experiments for various particle-volume ratios:

- Random initialisation of particles inside a 2 × 2 × 2 μm${}^{3}$ domain without boundaries;
- Random initialisation of particles inside a 2 × 2 × 2 μm${}^{3}$ domain with boundaries in *y* and *z* direction;
- Random initialisation of particles inside red blood cell.

The value 2 μm was chosen to reflect the thickness of RBC. For all of these we let the suspension relax with conservative forces turned on. We measured the minimal inter-particle distance and number of particle pairs that overlap, i.e., their distance is less than $2r_{h}$, Table 3. Here, we see that the boundaries have a significant effect. The same particle-volume fraction ϕ=0.545 results in no overlap in the small cubical domain without boundaries, but causes overlap of particles in domain with boundaries. Similarly, ϕ=0.503, which is low enough for all particles to relax in cubical domain with or without boundaries is high enough in RBC to cause 513 pairs of particles to overlap. The boundary effect means that there are larger particle-free areas next to boundary than in the middle of the suspension. The RBC with its particularly large surface (boundary) with respect to volume (that helps to transport oxygen in blood) means that the suspension needs to have a lower ϕ. Therefore, in the following, we will consider ϕ=0.5 and ϕ=0.485.

### 5.4. Considerations for Initial Values of Simulation Parameters

Given the typical ~8 μm size of an RBC, we decided to use slightly reduced radius of the sphere *R* = 3 μm to keep the volume of the sphere closer to that of an RBC. From Table 2 we see that to keep the correction term from boundary effects close to 10% we need to use ratio R/L less than 0.03 which gives us lower bound for box size *L* = 100 μm.

From Table 1 we can see that the viscosity of water and PBS solution ranges from 0.7 to 1.2 mPas, while the viscosity of blood plasma ranges from 1.5 to 2.2 mPas, depending on the temperature. We will use viscosity of suspending medium η = 1.2 mPas as a mid value.

We will work with the triangulated spheres with 304 nodes and RBCs with 1002 nodes. These triangulations give us the average edge length 0.66 μm for sphere with *R* = 3 μm and 0.4 μm for the RBC. The fluid in the lattice-Boltzmann method is discretized into a cubical lattice with grid size 1 μm.

### 5.5. Results

**Figure 5:**
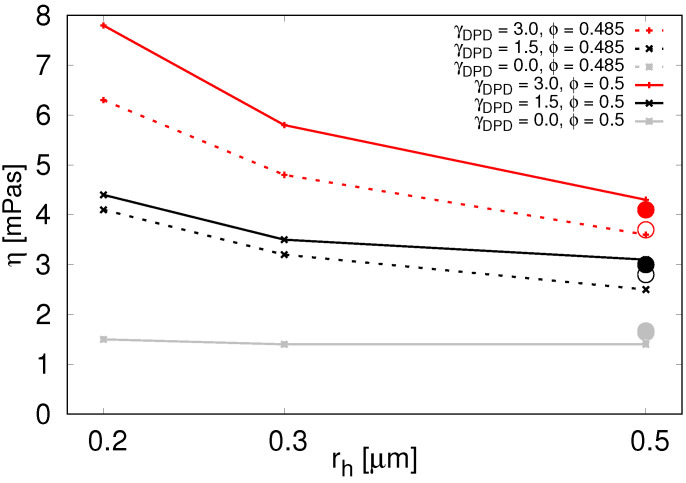
Viscosity for various particle radii and $\gamma_{DPD}$ (color coded) at ϕ=0.5 (solid lines) and ϕ=0.485 (dotted lines) in parallel plates computational experiment. For $\gamma_{DPD}=0$ (two gray lines that overlap) we used the soft-sphere interaction between the spherical particles. Data available in Table A5. The circles represent viscosity values from the drag force computational experiment with $r_{h}$ = 0.5 μm. Solid circles correspond to ϕ=0.5 and empty circles to ϕ=0.485. Two grey circles almost overlap.

When using the conservative forces alone, Figure 5 grey line, we see only very slight impact on the effective viscosity at volume fractions around ϕ=0.5. The particles rearrange quickly when needed and the desired higher values of viscosity cannot be achieved.

The inclusion of the dissipative forces effectively slows the particles and enables higher viscosity.

With the dissipative forces included, in Table 4 and Figure 5 we see that the parallel plate computational experiment and drag force computational experiment give very similar effective viscosity values at $r_{h}$ = 0.5 μm. Due to significantly larger number of particles needed for simulations with radii small enough to be relevant for inner RBC suspension, we performed only the parallel plate experiment with these, assuming there would be a similar correspondence to the drag force experiment. In the following, we summarize the results from the parallel plate experiment.

In Figure 5, we see that viscosity increases with decreasing particle radius. This is true for various values of $\gamma_{DPD}$ as well as for various particle volume densities. The second thing we see from Figure 5 is that viscosity increases with increasing $\gamma_{DPD}$. This means we can use the parameter $\gamma_{DPD}$ to tune the viscosity contrast.

Next, in Table 5, we see that for small shear rates there is almost no difference between viscosities measured at different shear rates. The shear-thinning properties of blood manifest at higher shear rates.

Furthermore, finally, there is a steep decline of viscosity for lower particle volume fractions as evidenced in Table 6. Volume fractions below 45% do not engage the particle dissipative forces enough to raise the viscosity sufficiently.

From these results, we conclude that it is possible to use various combinations of $\gamma_{DPD}$ and ϕ>0.45 to achieve the desired viscosity ratio. We list some of the suitable combinations in Table 7.

## 6. Model Validation

The primary motivation for using inner soft particles was to enable viscosity contrast in red blood cell model. Therefore, we next consider the following computational experiments with red blood cells with inner particle suspension: the gold standard optical tweezers experiment; both the stretching part to observe the elastic response of the membrane and the dynamic release part to observe the viscous response during relaxation.

### 6.1. Optical Tweezers-Stretching

We performed the computational stretching experiments similar to [49]. The elastic parameters of the RBC model and other details of the computational experiments were set according to recommendations from [18]. The final axial and transversal diameters of RBC with dissipative particles stretched with the applied force 0.192 nN (ϕ=0.485 and ϕ=0.5) were 14.2–14.25 μm and 4.28–4.54 μm, respectively. Data for other stretching forces are available in Table A6. For all stretching forces the diameters were within the biological data range. This was expected, as this is a static experiment and the particles have larger impact on the dynamics.

### 6.2. Optical Tweezers-Release

**Figure 6:**
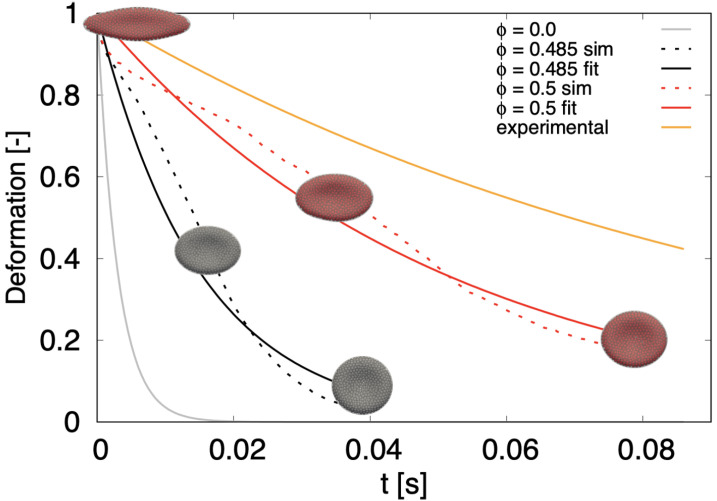
Relaxation of stretched RBC ($F_{stretch}$ = 0.192 nN) to discoid shape. Dotted lines correspond to simulations with particles, solid lines represent exponential fit, either to experimental data (orange) or to simulation data (gray, black and red). ϕ=0 stands for simulation without inner particles.

For the dynamical part of the optical tweezers computational experiment, we released the stretched cells and let them relax back to the original biconcave discoid shape. Similarly to [49,50], during the release we calculate the ratio

(7) $$\frac{\left(\lambda^{2}-1\right)\left(\lambda_{max}^{2}+1\right)}{\left(\lambda^{2}+1\right)\left(\lambda_{max}^{2}-1\right)},$$

where $\lambda =d_{a}/d_{t}$, $d_{a}$ is the axial diameter, $d_{t}$ is the transversal diameter and $\lambda_{max}$ represents the maximum deformation just before the release. In order to compare the return of the cell to the relaxed state, we fitted each time series (7) with an exponential curve $e^{-\frac{t}{t_{c}}}$ with characteristic time-constant $t_{c}$. The results are shown in Figure 6.

The grey line represents the relaxation of a simulated cell without dissipative particles (ϕ=0) with time constant $t_{c}^{\phi =0}=0.003$ s. The orange line represents data from biological experiment [50]. While the ranges given in the literature vary, the fastest release times shown here correspond to $t_{c}^{bio}=0.1$ s. The two dotted lines correspond to computational experiments with particles: black with ϕ=0.485 and red with ϕ=0.5. We see that the larger particle-volume fraction slows the return to relaxed shape more. The respective solid lines represent the exponential fit with $t_{c}^{\phi =0.485}=0.015$ s and $t_{c}^{\phi =0.5}=0.05$ s.

Although the relaxation time of the presented model is lower than time from the biological experiment, we see a significant shift towards the biological data compared to the previous model. Already in [50] the authors attribute the slow relaxation time to the viscosity of the RBC membrane. We expect that introducing phenomenological viscosity to membrane particles would further prolong the relaxation time towards the values measured in the biological experiment.

## 7. Discussion

In the present work, we have developed an RBC model that accounts for non-trivial viscosity ratio. We have analyzed the suspensions of dissipative particles that increase the viscosity of the suspending medium in two computational experiments: the parallel plate experiment and drag force experiment. The results of both overlapped and gave us the parameters of soft particle suspension that gives the desired inner cell viscosity. Using these parameters we have filled the membrane model of RBC with DPs and we tested this model. First we performed a static validation test—stretching of an RBC. Then we showed that inclusion of dissipative particles significantly reduced the discrepancy of the membrane RBC model in the computational release experiment. The relaxation time $t_{c}$ was increased from 0.003 s for the membrane model to 0.05 s for RBC model with inner particles, almost reaching biological RBC value of 0.1 s.

### 7.1. Model Limitations

Adding DPD particles has improved the performance of the model and achieved a slower relaxation of RBC to its original discoid shape. However, there are still possible improvements and limitations to the extent of its use.

We expect that introducing phenomenological viscosity to the membrane would slow down the relaxation time in the release experiment even further and thus achieve even better agreement with the biological data. The main limitations of our approach lie in the added computational complexity. The use of DPD particles requires using meshes with a larger number of nodes. This increases the computational time and thus this approach is less appropriate for modeling dense RBC suspensions.

The computational complexity is also the reason for performing drag force experiments only with small sized simulation boxes. We have assumed that since we achieved comparable results in the drag force experiment and the shear flow between two plates experiment, the results would be comparable also when using smaller particles in the drag force experiment. We estimated the effect of boundaries in a smaller box using simulations without DPD suspension and this allowed us to remove the effect from the simulation with DPD suspension. However, a more precise validation would have been achieved with the larger simulation box which would require at least an 8-fold increase in computational time. In our opinion the precision gain would not justify the computational time.

### 7.2. Novelty

The use of LBM for the inner and outer fluid and dissipative particles only for increasing the inner viscosity (as opposed to full DPD) leverages the LBM fixed lattice that is naturally suitable for parallelization and decreases computational time. The increase in inner viscosity is thus an emerging property without the need to solve equations to track the inner points at individual timesteps or detect them anew using ray-casting or similar methods.

The newly developed model proves to be more accurate while maintaining the computational complexity.

## Figures and Tables

**Table 1:** Viscosity measurements. Blood is a shear thinning fluid and thus the whole blood viscosity depends on shear rate. The values listed here approximately correspond to the shear rates 70–100 s${}^{-1}$.

	Temperature [${}^{\circ }$C]	Viscosity [mPa.s = cP]
	20	1.00
water	25	0.89
	37	0.69
	20	2.2 [41]
blood plasma	25	1.63 [42]
	37	1.5 [41]
	20	∼10 [43]
whole blood	25	∼7 [43]
	37	∼5 [44]
RBC cytosol	37	3–10 [15,45]
PBS	37	0.7 [46]

**Table 2:** Boundary effects—relative deviation of terminal velocity from theoretical value.

box size *L* [μm]	50	100	200	400
R/L[−]	0.06	0.03	0.015	0.0075
$v_{term}$ [mms${}^{-1}]$	4.59	5.24	5.63	5.79
boundary effect	−22.02%	−10.95%	−4.44%	−1.61%

**Table 3:** Determining the appropriate particle-volume fraction ϕ. $d_{min}$ (μm) is the minimal distance of two particles (over 1000 replications), $n_{pairs}$ is the maximum (over 1000 replications) number of particle pairs that are closer than $2r_{h}$, where $r_{h}$ = 0.2 μm.

Cube, No Boundaries			Cube, Boundaries			RBC		
ϕ	$d_{min}$	$n_{pairs}$	ϕ	$d_{min}$	$n_{pairs}$	ϕ	$d_{min}$	$n_{pairs}$
0.545	0.400	0	0.545	0.351	93	0.503	0.371	513
0.503	0.400	0	0.524	0.39994	3	0.485	0.389	40
0.461	0.400	0	0.503	0.400	0	0.467	0.400	0

**Table 4:** Comparison of effective viscosity [mPas] of particle suspensions in shear flow between parallel plates vs. in drag force experiment. The viscosity of the underlying fluid was 1.2 mPas, $\gamma_{DPD}$ = 3 nNm${}^{-1}$s and the radius of DPs was $r_{h}$ = 0.5 μm, shear rate given in $\left[s^{-1}\right]$.

	Parallel Plates		Drag Force Sphere	
ϕ	shear rate	$\eta_{in}$	shear rate	$\eta_{in}$
0.5	68	4.3	39	4.1
0.485	53	3.6	41	3.7
0.45	65	3.0	58	2.9

**Table 5:** At low shear rates, the shear rate has almost no impact on the particle suspension viscosity (here ϕ=0.5).

$r_{h}$	$\gamma_{\mathrm{DPD}}$	Shear Rate	$\eta_{\mathrm{in}}$
[μm]	[nNm${}^{-1}$s]	[s${}^{-1}$]	[mPas]
0.5	3.0	27	4.4
0.5	3.0	68	4.3
0.3	3.0	36	5.7
0.3	3.0	64	5.8
0.2	1.5	57	4.4
0.2	1.5	113	4.4

**Table 6:** Decrease in effective viscosity of particle suspension $\eta_{in}$ with respect to the decreasing volume fraction ϕ. Here $\gamma_{DPD}$ = 3 nNm${}^{-1}$s, radii given in μm.

	Viscosity [mPas]		
ϕ	$r_{h}=0.2$	$r_{h}=0.3$	$r_{h}=0.5$
0.5	7.8	5.8	4.3
0.485	6.3	4.8	3.6
0.45	4.7	3.9	3.0

**Table 7:** Parameters of the inner particle suspension for RBC that result in appropriate effective viscosity $\eta_{in}$.

ϕ	$r_{h}$	$\gamma_{\mathrm{DPD}}$	$\eta_{\mathrm{in}}$
[−]	[μm]	[nNm${}^{-1}$s]	[mPas]
0.5	0.2	2.5	6.0
0.5	0.3	3.4	6.0
0.485	0.2	2.75	6.0

## Data Availability

Data are contained within the article.

## Abbreviations

The following abbreviations are used in this manuscript:

RBC	Red blood cell
CFL	Cell free layer
LBM	Lattice-Boltzmann method
IBM	Immersed boundary method
IB-DC	Immersed boundary via dissipative coupling
DP	Dissipative particles
DPD	Dissipative particle dynamics

## Appendix A

**Table A1:** Simulation parameters, fluid.

LBM Grid [μm]	Time Step [μs]	Viscosity [mPas]	Density [kg/m${}^{3}$]
1	0.05	1.2	1

**Table A2:** Simulation parameters, elastic parameters of RBC in Section 6.1 and Section 6.2 and sphere in Section 5.2.2.

	RBC	Sphere
$k_{s}$ [μN/m]	0.009	2
$k_{b}$ [Nm]	0.007	2
$k_{al}$ [μN/m]	0.09	2
$k_{ag}$ [μN/m]	0.9	2
$k_{v}$ [N/m${}^{2}$]	0.9	2
no. of nodes	1002	304
mean edge length [μm]	0.4	0.66

**Table A3:** Simulation parameters, conservative interaction in the particle suspension.

Soft-Sphere		Hat
a	n	$F_{max}$
0.00032	1.5	0.6

**Table A4:** Simulation parameters, interaction of the particle suspension with the membrane. Outer interaction of suspension in the drag force experiment. Inner interaction of particle suspension with cell membrane from the inside, both drag force Section 5.2.2 and RBC experiments Section 6.1 and Section 6.2.

Interaction	a*	n*	$c_{\mathrm{soft}}^{*}$ [μm]
inner	0.02	1.5	$2r_{h}$
outer	0.128	1.5	$1.25r_{h}$

**Table A5:** Effective viscosity of particle suspensions in shear flow between parallel plates. The viscosity of the underlying fluid was 1.2 mPas. For $\gamma_{DPD}=0$ we used the soft-sphere interaction.

ϕ	$r_{h}\;[\mu$m]	$\gamma_{\mathrm{DPD}}$ [nNm${}^{-1}$s]	Shear Rate [s${}^{-1}]$	$\eta_{\mathrm{in}}$ [mPas]
0.5	0.5	0	53	1.4
0.5	0.3	0	70	1.4
0.5	0.2	0	83	1.5
0.5	0.5	1.5	86	2.8
0.5	0.3	1.5	71	3.5
0.5	0.2	1.5	57	4.4
0.5	0.5	3	68	4.3
0.5	0.3	3	64	5.8
0.5	0.2	3	65	7.8
0.485	0.5	0	53	1.4
0.485	0.3	0	70	1.4
0.485	0.2	0	85	1.5
0.485	0.5	1.5	77	2.5
0.485	0.3	1.5	84	3.2
0.485	0.2	1.5	98	4.1
0.485	0.5	3	53	3.6
0.485	0.3	3	56	4.8
0.485	0.2	3	56	6.3
0.45	0.5	3	65	3.0
0.45	0.3	3	70	3.9
0.45	0.2	3	64	4.7

**Table A6:** Final axial $d_{a}$ and transversal $d_{t}$ diameters in optical tweezers stretching experiment at volume fraction ϕ=0.5.

$F_{\mathrm{stretch}}$ [nN]	$d_{a}$ [μm]	$d_{t}$ [μm]
0	7.82	7.82
0.016	8.65	7.35
0.031	9.38	6.90
0.047	10.27	6.38
0.068	11.07	5.97
0.088	11.70	5.59
0.109	12.40	5.26
0.13	12.95	5.05
0.15	13.24	4.87
0.172	13.64	4.71
0.192	13.98	4.60
